# Case report: Malignant erythema in an ovarian cancer case: an uncommon paraneoplastic syndrome

**DOI:** 10.3389/fonc.2026.1593511

**Published:** 2026-03-16

**Authors:** Xiaolin Liu, Xingyu Wang, Lijie Wang

**Affiliations:** 1 Department of Obstetrics and Gynecology, Qilu Hospital, Shandong University, Jinan, Shandong, China; 2 Department of Ultrasound, The Fourth Affiliated Hospital of School of Medicine, and International School of Medicine, International Institutes of Medicine, Zhejiang University, Yiwu, China

**Keywords:** cytoreductive surgery, dermatomyositis, immunity, malignant erythema, ovarian cancer

## Abstract

Dermatomyositis is an autoimmune illness that affects the striated muscle and skin. It usually shows muscular and cutaneous manifestation. Age above 40 years and females are risk factors for malignant tumor in patients with dermatomyositis. In our case study, a woman with malignant erythema as her first symptom was reported. She was later diagnosed with ovarian cancer and underwent subtractive surgery, which caused the erythema to rapidly subside. Most similar DM cases suffered from muscular disorders including painless proximal symmetrical weakness as well as weakness of the pharyngeal muscle, often leading to aspiration pneumonia and dysphagia. Here, we reported this case with cutaneous symptoms in detailed clinical information including medicine use and clinical outcome. Cancer screening would be recommended in these intractable cases. On the other side, more researches are needed be focused on the immunity change of ovarian cancer.

## Introduction

Dermatomyositis is an autoimmune illness that affects the striated muscle and skin. It usually shows muscular and cutaneous manifestation. Age above 40 years and females are risk factors for malignant tumor in patients with dermatomyositis. In our case study, a woman with malignant erythema as her first symptom was reported. She was later diagnosed with ovarian cancer and underwent subtractive surgery, which caused the erythema to rapidly subside. Most similar DM cases suffered from muscular disorders including painless proximal symmetrical weakness as well as weakness of the pharyngeal muscle, often leading to aspiration pneumonia and dysphagia.

Here, we reported this case with cutaneous symptoms in detailed clinical information including medicine use and clinical outcome. Cancer screening would be recommended in these intractable cases. On the other side, more researches are needed be focused on the immunity change of ovarian cancer.

**Figure 1:**
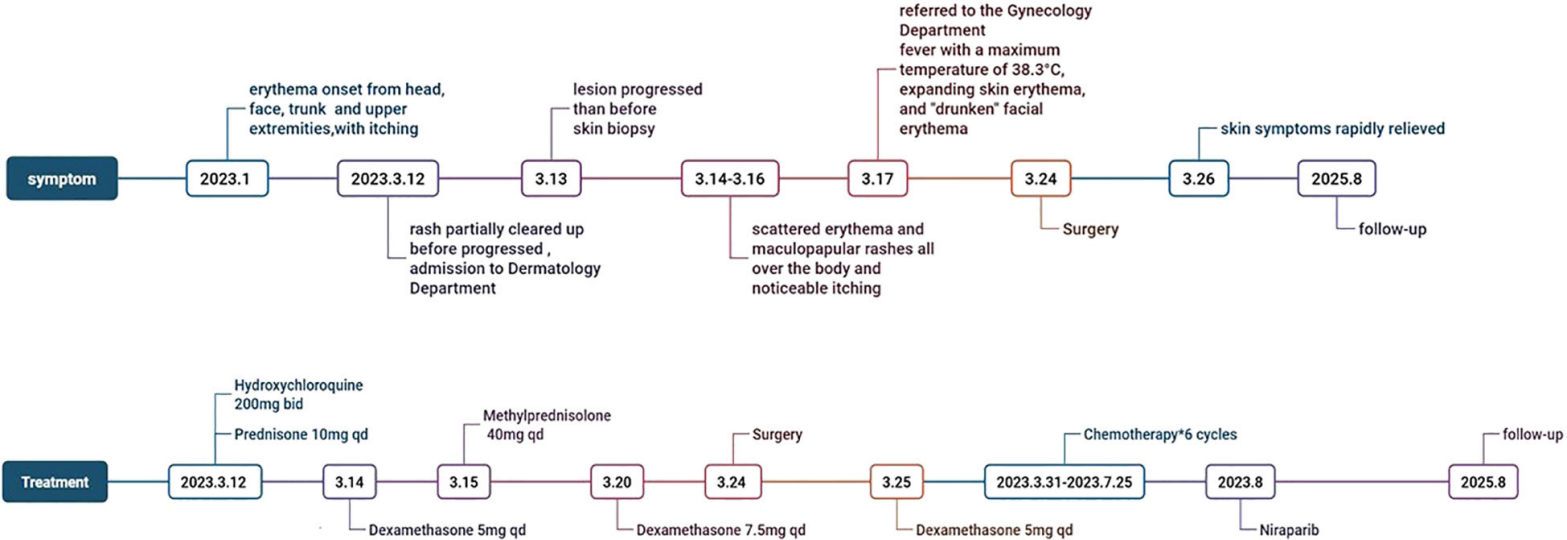
Symptom and treatment timeline.

A 68-year-old woman complaining of “erythema with itching on the head, face, trunk, and upper extremities for 2 months” visited the Department of Dermatology. Timetable was showed in **Figure 1**. The patient had erythema on her head, face, chest, V-zone, back of her hands and the extensor sides of both upper arms two months prior without any apparent cause. She also had edema in both of her eyelids, self-conscious itching, and sporadic water choking. Anti-Ro-52 and anti-nuclear antibodies were positive. Topical pimecrolimus ointment, hydroxychloroquine 200 mg bid, and prednisone 10 mg qd were administered. The patient noted that the rash had partially cleared up and her itching had subsided. The patient began to notice persistent erythematous itching on the top of his head three days ago. One of her sisters passed away from ovarian cancer.

A skin lesion sample was taken after the patient was admitted, and the postoperative pathology was connective tissue disease. Anomalies in the bilateral H-reflex were observed on electromyography. The blood test showed IgG 23.8g/L, IgE 592IU/mL, ESR 41mm/h and LDH 353U/L, listed in **Table 1**.

**Table 1:** Laboratory test results of the patient.

No.	Item	Result	Reference interval	Unit	Detection method
1	Anti-nuclear Antibody (ANA)	Positive	<1:100 Negative	–	Indirect Immunofluorescence
2	Anti-dsDNA Antibody	<10.00	0 - 100	IU/ml	Enzyme-Linked Immunosorbent Assay
3	Anti-Sm Antibody	<2.00	0 - 20	RU/ml	–
4	Anti-nucleosome Antibody (AnuA)	<2.00	0 - 20	RU/ml	Enzyme-Linked Immunosorbent Assay
5	Anti-ribosomal P Protein Antibody (FRNP)	<2.00	0 - 20	RU/ml	Enzyme-Linked Immunosorbent Assay
6	Anti-SSA Antibody	2.43	0 - 20	RU/ml	Enzyme-Linked Immunosorbent Assay
7	Anti-SSB Antibody	<2.00	0 - 20	RU/ml	Enzyme-Linked Immunosorbent Assay
8	Creatine Kinase (CK)	169	40 - 200	U/L	–
9	Alpha-Hydroxybutyrate Dehydrogenase (HBDH)	202.7	72 - 182	U/L	–
10	Creatine Kinase-MB Mass (CK-MB)	4.6	0.6 - 6.3	pg/ml	–
11	Myoglobin (MYO)	43.7	14.3 - 65.8	ng/ml	–
12	High-Sensitivity Cardiac Troponin I (HS-TnI)	3.2	0 - 17.5	pg/ml	–
13	Rheumatoid Factor (RF)	<11.20	0 - 20	KU/L	–
14	Anti-Myeloperoxidase Antibody (MPO)	1.2	0 - 20	RU/ml	Chemiluminescence
15	Proteinase 3 (PR3)	0.7	0 - 20	CU	Chemiluminescence
16	Anti-neutrophil Cytoplasmic Antibody - Perinuclear Pattern (pANCA)	Negative	<1:10 Negative	–	Indirect Immunofluorescence
17	Anti-neutrophil Cytoplasmic Antibody - Cytoplasmic Pattern (cANCA)	Negative	<1:10 Negative	–	Indirect Immunofluorescence
18	Anti-Glomerular Basement Membrane Antibody (GBM)	0.3	0 - 20	RU/ml	–
19	Humoral Immunity Panel Immunoglobulin G (IgG)	23.8	7 -016	g/l	–
20	Humoral Immunity Panel Immunoglobulin E1 (IgE1)	592	0 - 100	IU/ml	–
21	Lactate Dehydrogenase (LDH)	300	120-230	U/L	–

Muscle strength and tone are normal bilaterally. Hormones, vitamin C, cetirizine, and omeprazole were administered with poor results. The lesions on the head and face progressed worse than before, with scattered erythema and maculopapular rashes all over the body and noticeable itching. Computed tomography (CT) scan revealed an enlarged uterus with several hypodense shadows. Cystic solid tumor in the pelvis was detected by gynecologic ultrasonography (O-RADS: grade 5). CA125 level of 325U/ml. The patient was then referred to the gynecology department.

**Figure 2:**
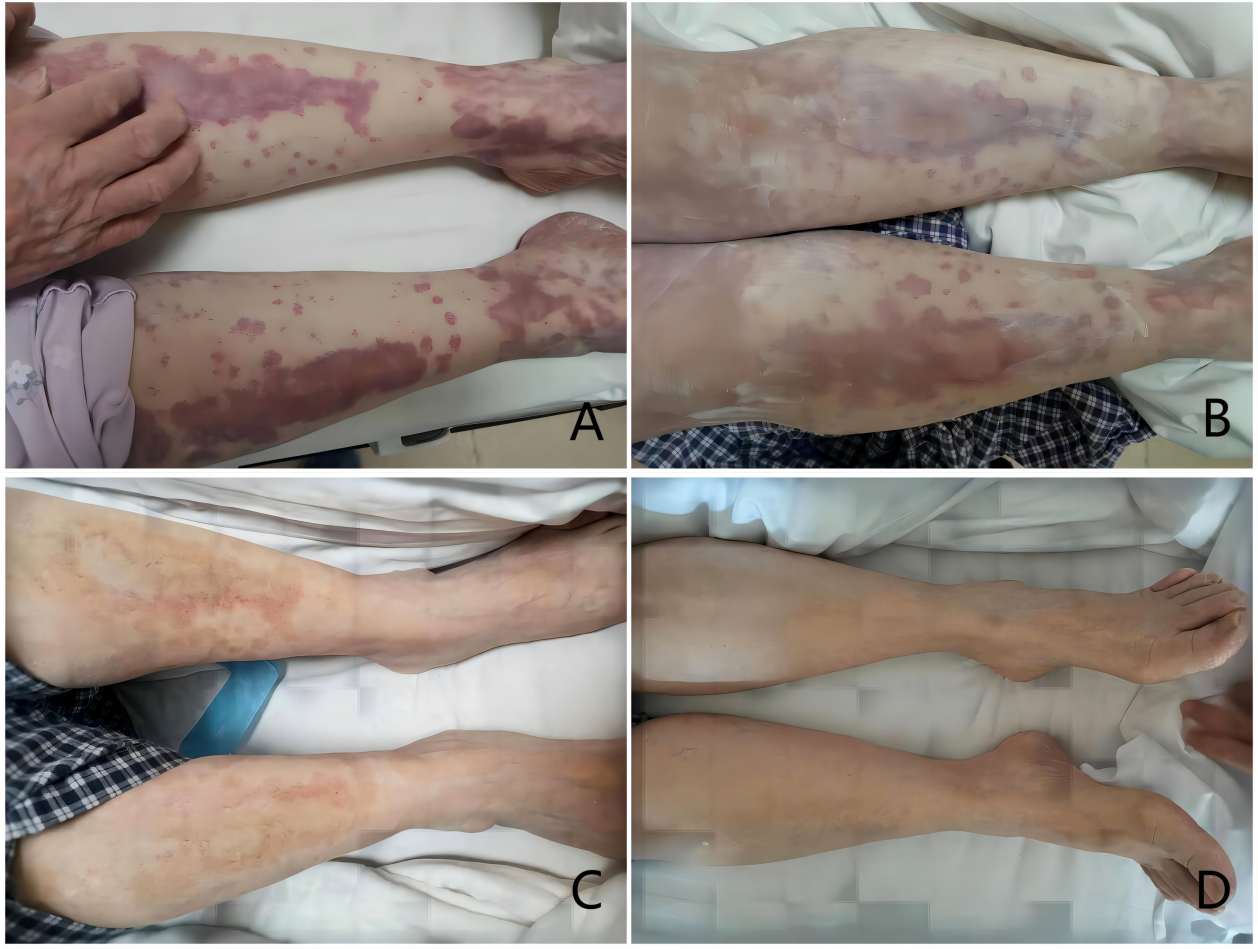
Changes in the patient’s rash (**(A)** 2023.3.21; **(B)** 2023.3.23; **(C)** 2023.3.26; **(D)** 2023.4.2 *Operation date: 2023.3.24).

The patient had a fever with a maximum temperature of 38.3 °C, expanding skin erythema, and “drunken” facial erythema on the day when she was referred to the gynecological department. An intense CT scan of the pelvic and abdominal cavity revealed cystic solid occupancy in the left adnexal region (maximum cross-sectional area: approximately 9.4x5.0 cm), which is thought to be ovarian cancer and has a limited, ill-defined demarcation between the uterus’ posterior wall and the nearby intestines. Multi-disciplinary treatment including rheumatology, anesthesiology, and dermatology was sought along with the patient’s medical history. The probable cause of malignant erythema was evaluated, and infectious causes like TROCH and EBV were ruled out. Gynecological surgery was recommended as soon as the skin symptoms had been controlled. The temperature and erythema were controlled after the glucocorticoid dosage was changed. The cytoreductive surgery was performed successfully. R0 was achieved. After the surgery, IgG 12.9g/L, IgE 582IU/mL, and ESR 14mm/h were measured and the skin symptoms were rapidly relieved. The changes were showed in **Figure 2**. Ovarian hypofractionated endometrioid adenocarcinoma, classified as stage IIB ovarian cancer, was the postoperative pathology. Chemotherapy of paclitaxel and carboplatin was administered. After 6 cycles of chemotherapy, the patient achieved clinical Complete Response of ovarian cancer. During the following 2 years of maintenance treatment with Niraparib, she showed no sign of recurrence of ovarian cancer, as well as the erythema. In the subsequent period, the patient underwent regular follow-up.

## Discussion

Ovarian cancer ranks the third most frequent malignant tumor of the female reproductive systems (1), following cervical and uterine body malignancy. In China, there were 27,200 fatal cases and 71,100 newly diagnosed cases of ovarian cancer in 2016 (2). Early-stage ovarian cancer typically has no noticeable symptoms, but in its latter stages, it may show up as abdominal fluid, bulk, distension, etc. The most typical symptom is acute onset of abdominal discomfort (3). Rarely, skin symptoms are presented as the initial sign of ovarian cancer. The patient in this case was initially diagnosed with skin connective tissue disease in the dermatology department with skin erythema as the primary symptom. The dermatologist remained cautious and discovered a pelvic tumor through a CT scan. Then the patient went to the gynecology department, where it was subsequently found that she had ovarian endometrioid adenocarcinoma of stage IIB.

The incidence of dermatomyositis (DM), an autoimmune illness with a rash and myopathy, is most prominent in people between the ages of 5–15 and 40 - 60. Typical skin lesions, such as Gottron papules, Gottron signs, and Heliotrope rash, are crucial for diagnosis and frequently come with itching or a burning discomfort (4). A myopathy is present in around 80% of DM patients, usually manifested as acute or subacute episodes of symmetrical proximal muscle weakness, either with or without increased muscle enzyme levels (5). The clinical progression of DM may not always resemble that of muscle disease (5). But in this case, the patient presented with a progressively worsening cutaneous symptom and mild muscle involvement. And in this way, it was a limitation that we did not perform the muscle biopsy and specific myositis antibodies test, including anti-TIF1γ, anti-NXP2, anti-Mi-2, anti-MDA-5 and anti-SAE antibodies.

Many malignancy-related cases of DM patients have been reported (6–8), as well as systematic reviews. After examination, some individuals with DM as the initial symptom may demonstrate recurrence indications, a poor response to immunological treatments, and the existence of hidden tumors (9). Critically, this case highlights the clinical observation that reinforces the link between DM and ovarian cancer: following cytoreductive surgery for ovarian endometrioid adenocarcinoma, which is also a kind of disease might be sensitive to immunity therapy, the patient’s cutaneous symptoms achieved significant improvement, with no sign of recurrence during follow-up. This finding is consistent with previous reports indicating that patients’ DM symptoms are relieved following tumor cytoreductive surgery (10), underscoring the pathogenic interplay between the autoimmune process and the underlying neoplasm. The resolution of the manifestations after surgery not only validates the diagnosis of cancer-associated DM but also suggests that tumor-derived antigens may have been driving the autoimmune response. Moreover, the absence of recurrence during follow-up further supports the efficacy of cytoreductive surgery in this case, emphasizing the importance of prompt tumor detection and intervention in DM patients with suspected malignancy.

The incidence of malignancy in patients with DM is approximately 10% (11). Breast cancer and ovarian cancer are the most common cancers in female DM patients. Additionally, females and age above 40 years are risk factors for combined malignancies in DM patients (12). Clinically, significant anti-TIF1 antibodies can help diagnose and prognosis disease outcomes in people with DM. In DM patients, some inflammatory cytokines (BAFF, sTNF-R1, sTNF-R2, etc.) may be indicators of malignancy (13). Combined DM in ovarian cancer patients has been hypothesized to be a prognostic factor for tumors (14). According to the risk categorization, all DM patients within 3 years of onset must undergo a cancer screening (15).

On the evidence of recent studies, the “second strike theory” has been put out to explain the etiology of cancer-associated DM. The “first strike” occurs when certain autoantibodies are produced in response to an infection, tumor, or damage. The “second strike” will initiate the dermatomyositis autoantigens’ upregulation and lead to an autoimmune response (16). Muscle cells and tumor cells may both express the same antigen, triggering a cross-immune reaction resulting in autoimmunity (16). Additionally, “foreign” antigens encoded by somatic mutations in particular tumor DNA and “wild-type” antigens found in particular tissues (such as skin, muscle, or viscera) might trigger cross-immune reactions (16). Additionally, tumor-infiltrating lymphocytes, PD-1/PD-L1 and CTLA-4 inhibitor pathways may contribute to the development of cancer-related dermatomyositis (16). In the context of this case, the resolution of DM symptoms post-cytoreductive surgery strongly suggests that the ovarian tumor was the primary driver of the autoimmune response, supporting the “second strike theory” and highlighting the clinical significance of targeting the underlying malignancy in managing cancer-associated DM.

## Conclusion

DM is an autoimmune disease with an incidence of malignancy of approximately 10%. The pathogenesis of cancer-associated DM is unclear. Malignancy treatment may alleviate symptoms of DM. Patients who have a primary diagnosis of dermatomyositis need to be alerted that an additional cancer may exist. Consequently, based on the risk categorization of DM patients, cancer screening is essential.

## Data Availability

The original contributions presented in the study are included in the article/supplementary material. Further inquiries can be directed to the corresponding author.
